# Associations Between Negative Social Experiences and Depressive Symptoms in Autistic Sexual and Gender Minority Youth

**DOI:** 10.1016/j.jaacop.2024.11.009

**Published:** 2025-02-20

**Authors:** Natalie Libster, Ryan E. Adams, Somer Bishop, Shuting Zheng, Julie Lounds Taylor

**Affiliations:** a Vanderbilt University Medical Center, Nashville, Tennessee; b Cincinnati Children’s Hospital Medical Center, Cincinnati, Ohio; c University of California, San Francisco, California

**Keywords:** autism, LGBTQ+, mental health, gender minority, social experiences

## Abstract

**Objective:**

Autistic lesbian, gay, bisexual, transgender, and queer/questioning (LGBTQ+) youth are at increased risk for negative mental health; however, no known studies have examined associations between specific social experiences and psychological distress within this group. The current study examined the effects of gender minority status and sexual minority status on negative social experiences (peer victimization and low degrees of authenticity) and depressive symptoms among autistic transition-aged youth, and explored whether associations between negative social experiences and depressive symptoms differed across gender/sexual identity.

**Method:**

Autistic youth (N = 203) between 15 and 26 years of age (mean = 18.69, SD = 2.58) were recruited through research registries. Youth and parents completed questionnaires, and youth participated in an interview. Biological sex, gender identity, and sexual orientation were collected from youth. Responses were coded into 3 gender/sexual identity groups: cisgender heterosexual (sex and gender match, heterosexual; n = 126), cisgender sexual minority (sex and gender match, sexual minority; n = 59), and gender minority (sex and gender do not match; n = 18). Youth questionnaires included measures of peer victimization, degree of authenticity when interacting with others, and depressive symptoms.

**Results:**

Peer victimization and authenticity did not differ across gender/sexual identity groups; however, gender minority youth reported greater depressive symptoms than cisgender heterosexual youth. Higher frequencies of peer victimization and lower degrees of authenticity were associated with depressive symptoms. The effects of peer victimization and authenticity on depressive symptoms were amplified for gender minority youth compared to cisgender heterosexual youth.

**Conclusion:**

The current study demonstrates how certain social experiences negatively affect the psychological well-being of autistic youth, especially those who identify as gender minorities.

## Plain language summary

LGBTQ+ youth with autism are at increased risk for negative mental health outcomes; however, no known studies have examined associations between specific social experiences and psychological distress within this group. In this study, measures of peer victimization, authenticity, and depressive symptoms were collected among youth with autism and compared across gender/sexual identity (cisgender heterosexual, cisgender sexual minority, and gender minority). Gender minority youth reported greater depressive symptoms than cisgender heterosexual youth, and peer victimization and authenticity had more negative effects on the depressive symptoms of gender minority youth compared to cisgender heterosexual youth. This study demonstrates how key social experiences may negatively impact the psychological well-being of youth with autism, especially those who identify as gender minorities. It also highlights the need for early intervention to support the mental health of these high-risk youth.

## Graphical abstract

**Figure:**
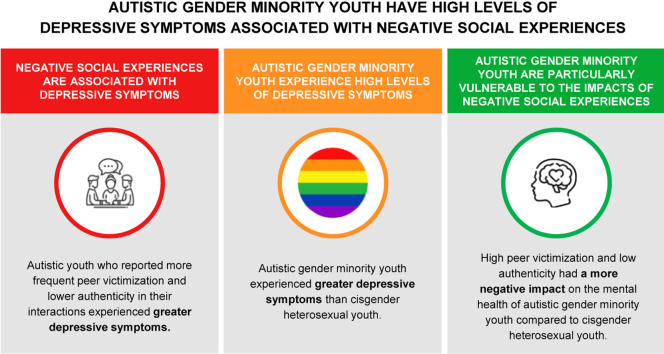

Autistic youth and those who identify as lesbian, gay, bisexual, transgender, and queer/questioning (LGBTQ+) are at increased risk for experiencing negative mental health outcomes, including depression and suicidal ideation.^1^^,^^2^ These 2 identities often intersect, as greater gender and sexual orientation diversity is found in the autistic population than in the general population.^3^, ^4^, ^5^, ^6^ Prior studies have found that autistic LGBTQ+ adolescents and adults experience poorer mental health than autistic non-LBGTQ+ and nonautistic LGBTQ+ individuals.^6^, ^7^, ^8^, ^9^ However, there has been little research into why autistic LGBTQ+ youth are at even greater risk for poor mental health than are their non-LGBTQ+ autistic peers.

The double minoritized identities of autistic LGBTQ+ youth may place them at especially high risk for negative social experiences and the associated mental health consequences. Both autistic youth and LGBTQ+ youth experience higher rates of peer victimization,^10^^,^^11^ and more frequent victimization among these youth is associated with greater internalizing symptoms.^12^, ^13^, ^14^ Although autism diagnostic status and LGBTQ+ status independently increase the likelihood of these youth to experience peer victimization, no studies, to our knowledge, have examined the peer victimization experiences of autistic LGBTQ+ youth.

Furthermore, autistic LGBTQ+ youth may not always feel safe being their authentic selves around others. In the current study as well as others,^15^ authenticity is defined as the alignment between one’s self-expression and one’s internal sense of self; in other words, authenticity is the feeling that “one is being their real self.”^16^ LGBTQ+ youth who do not feel comfortable being their authentic selves around others may choose to conceal their gender and sexual identities. Identity disclosure is associated with improved psychological well-being among LGBTQ+ adults,^17^^,^^18^ yet many LGBTQ+ youth face challenges in disclosing their gender and sexual identities due to fear of rejection and stigma.^19^, ^20^, ^21^ Furthermore, autistic youth who do not feel comfortable being their authentic selves around others may suppress or “camouflage” their autistic traits. These camouflaging behaviors are associated with reduced psychological well-being among autistic adults^22^^,^^23^; yet many autistic individuals, especially those who are female, suppress their autistic traits to be perceived as “normal” and to be accepted by their nonautistic peers.^23^^,^^24^ Given the hesitations of LGBTQ+ youth to disclose their identities and the tendencies of autistic youth to camouflage their autistic traits, autistic LGBTQ+ youth may be at heightened risk for experiencing low authenticity around other people, leading to poor mental health outcomes in this group.

In addition, LGBTQ+ status may further amplify the impact of peer victimization and authenticity on depressive symptoms in autistic youth. Although this hypothesis has not been examined in autistic samples, prior research has shown that in the general population, peer victimization has a more negative impact on the mental health of LGBTQ+ youth relative to non-LGBTQ+ youth.^25^ For example, a study by Espelage *et al.*^25^ found that high school students who were more frequently victimized experienced higher rates of suicidality than those less frequently victimized, regardless of LGBTQ+ status; however, as peer victimization became more frequent, rates of suicidality increased more rapidly among LGBTQ+ youth compared to non-LGBTQ+ youth. Autistic LGBTQ+ youth may therefore not only experience more frequent victimization and lower degrees of authenticity than autistic non-LGBTQ+ youth, but these social experiences may have an amplified negative impact on their mental health compared to that of autistic non-LGBTQ+ youth.

Research on LGBTQ+ individuals in autistic and nonautistic samples often combine cisgender sexual minorities and gender minorities into a single LGBTQ+ group^7^^,^^26^; however, research on LGBTQ+ youth in the general population suggest that mental health outcomes vary across different aspects of LGBTQ+ identity.^27^ Therefore, in the current study, autistic youth with gender minority identities and with cisgender sexual minority identities were distinguished. The current study extends prior work demonstrating poor mental health among autistic LGBTQ+ adolescents and adults^7^, ^8^, ^9^ by asking the following 3 questions: (1) Do autistic gender minority and cisgender sexual minority youth experience more frequent peer victimization and lower degrees of authenticity than autistic non-LGBTQ+ youth? (2) Are gender minority status, sexual minority status, peer victimization, and authenticity associated with depressive symptoms among autistic youth? (3) Are the effects of peer victimization and authenticity on depressive symptoms amplified for autistic gender minority and cisgender sexual minority youth relative to autistic non-LGBTQ+ youth?

## Method

### Participants

The current sample consisted of 203 autistic youth between the ages of 15 and 26 years (mean = 18.69, SD = 2.58) who participated in a larger study focused on mental health among autistic youth (Table 1 lists demographic characteristics of the sample). Participants were recruited from research match registries hosted by the Simons Simplex Collection (SSC)^28^ and the Simons Foundation Powering Autism Research for Knowledge (SPARK),^29^ as well as from clinical research research registries at the authors’ institutions. For the larger study, eligibility criteria included youth with the following characteristics: (1) age 15 to 26 years at the time of the study; (2) having received an autism diagnosis before the age of 18 years; (3) having a historic IQ or parent-report IQ of 70 or above (this is the standardized IQ cut-off for intellectual disability and was a proxy for self-report ability)^30^; and (4) being able to self-report and consent independently (assent for youth <18 years of age). Youth from the SSC and clinical research registries received clinician-administered diagnostic evaluations to confirm their autism diagnoses, whereas parents of youth from the SPARK registry reported their children’s autism diagnoses (note that parent-reported diagnoses of autism are highly valid).^29^ All diagnoses were based on *DSM-5* criteria. Although youth were primary respondents, we also asked parents/legal guardians to fill out an online survey. Youth over the age of 18 years were able to participate without a legal guardian; however, in nearly all cases (n = 197), a parent/legal guardian (183 mothers) participated in data collection. Although 241 youth/parent dyads participated in data collection for the larger study, 34 youth did not report their sexual identities, and 4 youth did not complete the outcome measures needed for the current analysis. These participants were therefore not included in the present sample.

**Table 1:** Demographic and Behavioral Characteristics of the Sample

Descriptive	Frequency	%
Gender		
Male	152	74.9
Female	41	20.2
Nonbinary	10	4.9
Biological sex		
Male	154	75.9
Female	49	24.1
Gender minority status		
Cisgender	185	91.1
Gender minority	18	8.9
Race		
American Indian	6	2.9
Asian	10	4.9
Black or African American	8	3.9
White	174	85.3
Other	3	1.5
Current educational status		
In high school	124	61.1
In post**–**secondary education program	37	18.2
Not in educational program	40	19.7
SRS-2 total *t* score		
Normal (≤59)	40	19.7
Mild (60-65)	38	18.7
Moderate (66-75)	72	35.5
Severe (≥76)	53	26.1
	Mean (SD)	Range
Age	18.69 (2.58)	15-26
IQ standard score	100.93 (16.50)	68-160
SRS-2 total *t* score	68.71 (11.51)	41-107

Note: SRS-2 = Social Responsiveness Scale**–**2.

### Procedures

The SSC and SPARK registries used information from their databases (collected during initial registration) to identify families that met the inclusion criteria for the study. Each registry shared the study information with eligible families via e-mail, and if families expressed interest in participating, their information was passed on to the study team. Families from clinical research registries who met inclusion criteria for the study and who had agreed to be contacted for future research opportunities were sent an e-mail by project staff with the study information. The study team then reached out to families who agreed to be contacted about the study via telephone or video call. To confirm that youth were able to self-report, the study team verbally administered a short series of questions that assessed their language proficiency.^31^ Eligible participants and their parents provided consent (assent for youth <18 years of age) to the study during this phone/video call, after which youth and parents completed the remotely administered study protocol. The Institutional Review Boards at the authors’ institutions, as well as a community advisory committee from SPARK, approved all study procedures.

### Measures

#### Sexual and Gender Identity

Self-reports of biological sex (male, female) and gender (male, female, nonbinary/other) were completed by the youth via questionnaire. Youth were further asked to indicate whether they were sexually attracted to the following: mostly or always men; mostly or always women; both men and women to a similar degree; other/neither; or prefer not to respond. Responses were coded into 1 of 3 gender/sexual identity groups: (1) cisgender heterosexual (n = 126; 84% biological male); (2) cisgender sexual minority (n = 59; 70% biological male); and (3) gender minority (n = 18; 39% biological male). Youth were coded as cisgender if their gender identity matched their biological sex, and as gender minority if their gender identity did not match their biological sex. Cisgender youth were further coded as sexual minority if they reported being attracted to partners of the same gender (ie, homosexual), partners of both the same and opposite gender (ie, bisexual), or partners with other gender identities. Sexual orientations were coded based on definitions provided by the Centers for Disease Control and Prevention (CDC), which describe sexual attraction according to gender identity.^32^ Frequencies of biological sex, gender identity, and gender of preferred sexual partner across the 3 gender/sexual identity groups are presented in Table S1, available online.

#### Peer Victimization

Youth completed the Revised Schwartz Peer Victimization Scale (RSPVS),^33^ an 18-item questionnaire containing descriptions of various types of peer victimization. Participants indicated how often they experienced each type of victimization (eg, “How often do others call you names?”) using a 7-point scale ranging from 1 (“Never happens to me”) to 7 (“Happens to me almost every day”). Ratings were then averaged across all 18 items to produce a mean victimization score, with higher scores indicating more frequent victimization. An alpha of 0.93 was found for the current sample.

#### Authenticity

Youth were asked to rate their degree of authenticity when interacting with others on 1 of the items on the Autism Spectrum Quality of Life Scale (ASQoL).^34^ The ASQoL is a 9-item questionnaire administered alongside the World Health Organization Quality of Life Scale^35^ to assess aspects of quality of life that may be especially relevant to autistic populations. Respondents are given the following prompt: “Please think about your life in the last 2 weeks and answer the following questions.” To examine the effect of authenticity, the current analyses focused on Item #2 on the ASQoL: “Can you ‘be yourself’ around your friends/people you know well? For example, you don't have to put on an ‘act’.” Response options included 1 (“Not at all”), 2 (“A little”), 3 (“Moderately”), 4 (“Mostly”) and 5 (“Totally”). The item score was included in the analyses as a proxy indicator for authenticity.

#### Depressive Symptoms

Youth completed the Beck Depression Inventory**–**II (BDI-II),^36^ a 21-item questionnaire that measures the presence and severity of symptoms of depression. Ratings were summed across all of the items to produce a total score, with higher scores indicating greater depressive symptoms. Research has shown that the BDI-II is a valid measure of depressive symptoms in autistic samples.^37^ An alpha of 0.92 was found for the current sample.

#### Covariates

Youth reported their date of birth (used to calculate age) via questionnaire. For 72 youth, IQ scores from standardized cognitive assessments were available from the datasets of their respective research registries. The remaining youth (n = 126) were administered the Peabody Picture Vocabulary Test**–**5 (PPVT-5)^38^ during an online interview to provide an estimate of cognitive ability.^39^ Furthermore, parents completed the Social Responsiveness Scale**–**2 (SRS-2),^40^ a 65-item questionnaire that measures the severity of social communication difficulties and restrictive/repetitive behaviors associated with autism. Parents rated the degree to which each behavior described their child (eg, “Would rather be alone than with others”) on a 4-point scale ranging from 0 (“Not true”) to 3 (“Almost always true”). The raw scores on the SRS-2 were converted into *t* scores, which were used in the current study.

### Data Analysis

To address our first research question, 2 separate linear regression models were conducted to examine whether gender minority status and sexual minority status were associated with peer victimization frequency and feelings of authenticity. To address our second research question, a third linear regression model was conducted to determine whether gender minority status, sexual minority status, peer victimization, and authenticity were associated with depressive symptoms. In all 3 regression models, levels of peer victimization, authenticity, and depressive symptoms among cisgender sexual minority youth and gender minority youth were compared to those of cisgender heterosexual youth. Finally, to address our third research question, 2 moderation models were implemented to examine whether the effects of peer victimization and authenticity on depressive symptoms differed across cisgender heterosexual, cisgender sexual minority, and gender minority youth. Because age, IQ, and autism symptomology are associated with peer victimization^12^^,^^41^^,^^42^ and because biological sex is associated with depressive symptoms^43^ among autistic youth, we controlled for these variables in the models. The linear regression analyses were implemented using R version 4.1.0,^44^ and the moderation analyses were implemented using PROCESS version 4.0.^45^

To aid interpretability, we treated the authenticity variable as continuous rather than ordinal in all analyses. Ordinal variables with 5 or more categories can almost always be treated as continuous without altering the model estimates.^46^, ^47^, ^48^ Of the 203 youth who reported on their sexual and gender identities and who completed the outcome measures needed for the current analysis, 5 participants (2.5%) were missing historic IQ information and were not administered the PPVT-5, and 6 participants (3.0%) were missing SRS-2 scores. The mean IQ (100.9) and SRS-2 *t* score (68.7) of the sample were imputed for the missing cases. There were no missing data on the other covariate measures for participants included in this analysis.

## Results

Our first research question examined whether autistic gender minority youth and sexual minority youth experienced more frequent peer victimization and lower degrees of authenticity than autistic non-LGBTQ+ youth. Model estimates are presented in Table 2. Peer victimization frequency among cisgender sexual minority and gender minority youth did not differ from that in cisgender heterosexual youth. Similarly, feelings of authenticity did not differ among cisgender sexual minority and gender minority youth compared to cisgender heterosexual youth (Table 2). In general, youth in the current sample reported low levels of peer victimization (mean = 1.72, between 1 [“Never happens to me]” and 2 [“Once a year”]) and high levels of authenticity (median = 4, corresponding to “Mostly can be yourself around friends/people you know well”). Albeit not a primary aim of the study, we found that youth who were younger experienced more frequent peer victimization than those who were older, and youth with greater autism symptomology experienced more frequent peer victimization and lower authenticity than those with less autism symptomology.

**Table 2:** Model Estimates Predicting Peer Victimization and Authenticity

Outcome	Predictor	Unstandardized estimate	95% CI	*p*	*R*^*2*^
Peer victimization					0.09
	Gender/sexual identity				
	Cisgender heterosexual^a^	—	—	—	
	Cisgender sexual minority	0.24	–0.05 to 0.53	.10	
	Gender minority	0.41	–0.07 to 0.89	.10	
	Biological female (vs male)	–0.02	–0.33 to 0.30	.92	
	Age	–0.05	–0.10 to –0.01	.04	
	IQ	0.01	–0.01 to 0.01	.08	
	Autism symptomology	0.01	0.01 to 0.02	.04	
Authenticity					0.04
	Gender/sexual identity				
	Cisgender heterosexual^a^	—	—	—	
	Cisgender sexual minority	0.03	–0.30 to 0.37	.85	
	Gender minority	0.02	–0.55 to 0.58	.95	
	Biological female (vs male)	–0.09	–0.46 to 0.28	.63	
	Age	–0.01	–0.07 to 0.05	.81	
	IQ	–0.01	–0.02 to 0.01	.15	
	Autism symptomology	–0.02	–0.03 to –0.01	.03	

**Note:**

a Reference group.

Our second research question examined whether gender minority status, sexual minority status, peer victimization, and authenticity were associated with depressive symptoms among autistic youth. Model estimates are presented in Table 3. Gender minority youth experienced greater depressive symptoms compared to cisgender heterosexual youth; however, depressive symptoms did not differ between cisgender heterosexual and cisgender sexual minority youth. In the current sample, 66.7% of autistic gender minority youth (12 of 18) had “moderate” (scores of 19-29) or “severe” (scores >29) depressive symptoms as classified on the BDI. This was over twice the rate of moderate or severe depressive symptoms observed among cisgender sexual minority youth in this sample (18 of 59 = 30.5%) and over 4 times the rate observed among autistic youth who did not identify as a gender or sexual minority (18 of 126 = 14.3%). Higher self-reported peer victimization and lower authenticity were also associated with greater depressive symptoms (Table 3, model 1). Albeit not a primary aim of the study, we found that youth who were biologically female experienced greater depressive symptoms than those who were male.

**Table 3:** Estimates Predicting Depressive Symptoms in Regression and Moderation Models

Predictor	Model 1			Model 2			Model 3		
	Unstandardized estimate	95% CI	*p*	Unstandardized estimate	95% CI	*p*	Unstandardized estimate	95% CI	*p*
Age	0.22	–0.26 to 0.69	.37	0.26	–0.24 to 0.76	.31	0.11	–0.37 to 0.58	.65
Biological female (vs male)	3.73	0.75 to 6.72	.01	4.19	1.00 to 7.38	.01	4.29	1.22 to 7.36	.01
IQ	0.05	–0.02 to 0.13	.16	0.08	–0.01 to 0.16	.05	0.07	–0.01 to 0.14	.08
Autism symptomology	0.05	–0.06 to 0.16	.34	0.10	–0.01 to 0.22	.07	0.10	–0.01 to 0.21	.08
Gender/sexual identity									
Cisgender heterosexual^a^	—	—	—	—	—	—	—	—	—
Cisgender sexual minority	1.99	–0.75 to 4.72	.15	–2.49	–8.48 to 3.50	.41	–5.60	–16.55 to 5.36	.32
Gender minority	9.36	4.79 to 13.94	<.001	–0.31	–10.54 to 9.91	.95	24.80	10.22 to 39.37	.001
Peer victimization	2.60	1.26 to 3.94	<.001	1.76	–0.19 to 3.71	.08	—	—	--
Authenticity	–3.29	–4.44 to –2.15	<.001	—	—	—	–3.67	–5.13 to –2.21	<.001
Victimization∗gender/sexual identity									
Victimization∗cisgender heterosexual^a^	—	—	—	—	—	—	—	—	—
Victimization∗cisgender sexual minority	—	—	—	2.45	–0.53 to 5.44	.11	—	—	—
Victimization∗gender minority	—	—	—	4.75	0.33 to 9.17	.04	—	—	—
Authenticity∗gender/sexual identity									
Authenticity∗cisgender heterosexual^a^	—	—	—	—	—	—	—	—	—
Authenticity∗cisgender sexual minority	—	—	—	—	—	—	2.05	–0.62 to 4.73	.13
Authenticity∗gender minority	—	—	—	—	—	—	–3.83	–7.46 to –0.20	.04
*R*^*2*^	.37			.29			.35		

**Note:**

a Reference group.

**Figure 1:**
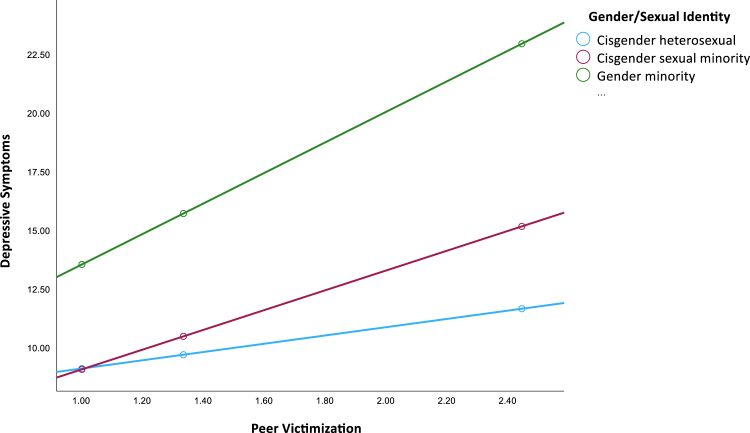
Moderation Results Depicting Effects of Peer Victimization on Depressive Symptoms Across Gender/Sexual Identity Groups

Our third research question examined whether the effects of peer victimization and authenticity on depressive symptoms differed for autistic gender minority youth and sexual minority youth relative to autistic non-LGBTQ+ youth. The moderation model detected a statistically significant interaction between peer victimization and gender minority identity (Table 3, model 2). Youth who were more frequently victimized reported greater depressive symptoms regardless of gender minority status; however, the association between peer victimization and depressive symptoms was stronger among gender minority youth (*b* = 6.51, 95% CI [2.53, 10.50], *p* = .002) compared to cisgender heterosexual youth (*b* = 1.76, 95% CI [−0.19, 3.71], *p* = .08). This interaction is depicted in Figure 1. As can be seen in the figure, gender minority youth reported greater depressive symptoms than did cisgender heterosexual youth, regardless of how frequently they were victimized. However, as peer victimization became more frequent, depressive symptoms increased more rapidly among gender minority youth compared to cisgender heterosexual youth. There was no significant interaction between peer victimization and cisgender sexual minority identification (Figure 1).

**Figure 2:**
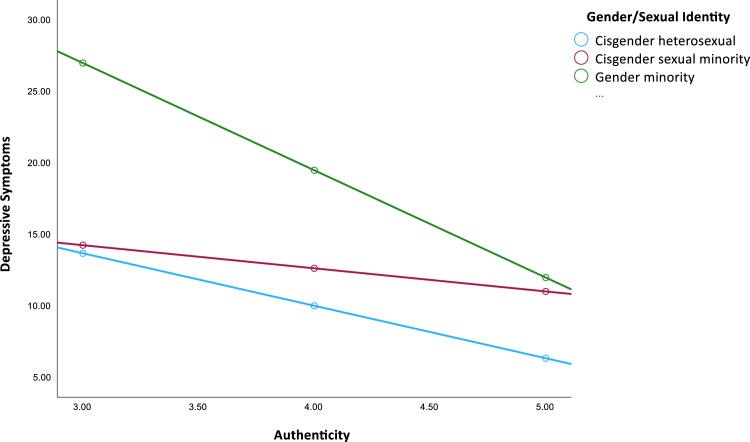
Moderation Results Depicting Effects of Authenticity on Depressive Symptoms Across Gender/Sexual Identity Groups

We also detected a statistically significant interaction between authenticity and gender minority identification (Table 3, model 3). Youth who reported lower authenticity experienced greater depressive symptoms, regardless of gender minority status; however, the association between authenticity and depressive symptoms was stronger among gender minority youth (*b* = −7.50, 95% CI [−10.79, −4.21], *p* < .001) compared to cisgender heterosexual youth (*b* = −3.67, 95% CI [−5.13, −2.21], *p* < .001). This interaction is depicted in Figure 2. As can be seen in the figure, gender minority youth experienced greater depressive symptoms than did cisgender heterosexual youth, regardless of their degree of authenticity. However, as youth reported greater authenticity in their interactions, depressive symptoms decreased more rapidly among gender minority youth compared to cisgender heterosexual youth. There was no significant interaction between authenticity and cisgender sexual minority identification (Figure 2).

## Discussion

This study not only provides further evidence of increased rates of depressive symptoms in autistic gender minority youth,^8^^,^^9^ but it is also is the first study to explore how negative social experiences affect the mental health of autistic LGBTQ+ youth relative to autistic non-LGBTQ+ youth. Although there were few gender minority youth in the current sample relative to cisgender heterosexual youth, the rate of gender diversity in the sample (8.9%) was similar to those found in other samples of autistic adolescents (6.5%)^6^ and adults (3.9%-11.4%).^4^^,^^6^ Meanwhile, a recent report found the prevalence of gender dysphoria among adults in the general population to be as high as 5%.^49^ Not only is there greater gender diversity among autistic youth compared to that in the general population, but autistic gender minority youth are at exceptionally high risk for experiencing depressive symptoms. It is therefore imperative that the mental health needs of these individuals are well understood and addressed.

Autistic gender minority youth and sexual minority youth reported similar degrees of peer victimization and authenticity as did autistic non-LGBTQ+ youth. It is important to note that youth in the current sample generally reported low levels of peer victimization and high levels of authenticity. The low frequencies of peer victimization in the sample are likely attributed to the age range of participants; peer victimization in the general population tends to peak in early-to-mid adolescence and then decrease,^50^ whereas the ages in the current sample ranged from late adolescence to early adulthood (and younger age was associated with more frequent peer victimization). Differences in peer victimization across gender/sexual identity may therefore be more noticeable among younger autistic adolescents or autistic youth who experience higher rates of peer victimization.

Although peer victimization and authenticity did not differ across gender/sexual identity, both higher frequencies of peer victimization and lower degrees of authenticity had an amplified negative impact on the mental health of gender minority youth compared to cisgender heterosexual youth. Gender minority youth in the general population are often victimized because of their gender identities,^51^ and conceal their gender identities from others because of fear of rejection^20^; however, in the present study, we were unable to investigate why autistic gender minority youth may have been victimized or why they may have lacked authenticity in their interactions. Autistic gender minority youth who are victimized because of their gender identities and/or who feel uncomfortable embracing their gender identities because of perceived stigma may be especially negatively affected by these experiences, even if they experience the same amount of victimization and authenticity as autistic cisgender youth. Thus, an area of future research is to examine whether the perceived stigma, harassment, and discrimination based on one’s gender identity place autistic gender minority youth at higher risk for depressive symptoms compared to autistic cisgender youth.^27^

There are some limitations to this study that are worth noting. We chose the item on the Autism Spectrum Quality of Life Scale (ASQoL) as a measure of authenticity because of its high face validity; however, single item measures lack precision and raise concerns around measurement error. Thus, the current findings around authenticity should be interpreted with caution until future studies using more psychometrically sound measures of authenticity can replicate the current findings. The majority of studies examining identity disclosure in LGBTQ+ samples have been qualitative,^52^ and although measures have been used in autistic samples to examine the degree to which respondents conceal or mask their autistic behaviors,^53^ these measures were not specifically designed to assess authenticity. More research is therefore needed to improve the measurement of authenticity.

There were also limitations of the measure used to determine sexual orientation, which asked respondents to indicate the gender of their preferred sexual partner. Although we were able to use this measure to determine the sexual orientations of cisgender youth, this measure was not methodologically appropriate for determining sexual orientation among gender minority youth. Asking gender minority youth to label their sexual orientations has been found to be the best practice for determining their sexual identities.^27^ Prior research has also found that gender minority youth with sexual minority identities in the general population experience higher rates of anxiety and depressive symptoms than gender minority youth with heterosexual identities.^27^ Therefore, additional research is needed to assess whether depressive symptoms among autistic gender minority youth differ based on sexual minority status.

Finally, the current study had limitations regarding the generalizability of the findings. The autistic youth in the current study all had IQ scores above 70 and were able to report on their gender and sexual identities, social experiences, and mental health; these findings may not be generalizable to autistic youth who have intellectual disability or are otherwise unable to self-report. The current sample was also recruited from existing research registries and had limited racial/ethnic diversity; understanding the social experiences and mental health outcomes of autistic LGBTQ+ youth in racial/ethnic minority groups is an important area of future research.

The exceptionally high levels of depressive symptoms among autistic gender minority youth in the current study highlight the immediate need for mental health interventions in this subpopulation. Certain social experiences, including high frequencies of peer victimization and low degrees of authenticity, seem to have a particularly negative impact on the mental health of autistic gender minorities. Autistic gender minority youth may therefore benefit from interventions, such as participation in autism LGBTQ+ support groups, that provide a safe space for them to be their authentic selves without fear of being victimized. Overall, it is important that future interventions promote social experiences in which autistic youth, and especially autistic gender minority youth, are free of victimization and can embrace their identity.

## CRediT authorship contribution statement

**Natalie Libster:** Conceptualization, Formal analysis, Writing – original draft. **Ryan E. Adams:** Writing – review & editing, Supervision, Project administration. **Somer Bishop:** Writing – review & editing, Supervision, Project administration, Funding acquisition. **Shuting Zheng:** Writing – review & editing, Supervision, Data curation. **Julie Lounds Taylor:** Conceptualization, Funding acquisition, Project administration, Supervision, Writing – review & editing.
