# Supplementary material for: Associations Between Negative Social Experiences and Depressive Symptoms in Autistic Sexual and Gender Minority Youth
Source: JAACAP Open. 2025 Feb 20;3(4):1006–15. doi: 10.1016/j.jaacop.2024.11.009 (PMC12684457; doi:10.1016/j.jaacop.2024.11.009)
Supplement: Supplemental Table S1 [file mmc1.docx]

|  | Cisgender heterosexual (n, %) | Cisgender sexual minority  (n, %) | Gender minority (n, %) |
| --- | --- | --- | --- |
| Biological sex |  |  |  |
| Male | 106 (84.1%) | 41 (69.5%) | 7 (38.9%) |
| Female | 20 (15.9%) | 18 (30.5%) | 11 (61.1%) |
| Gender identity |  |  |  |
| Male | 106 (84.1%) | 41 (69.5%) | 5 (27.8%) |
| Female | 20 (15.9%) | 18 (30.5%) | 3 (16.7%) |
| Non-binary/other | 0 | 0 | 10 (55.6%) |
| Gender of preferred sexual partner |  |  |  |
| Mostly or always men | 20 (15.9%) | 6 (10.2%) | 2 (11.1%) |
| Mostly or always women | 106 (84.1%) | 5 (8.5%) | 2 (11.1%) |
| Both men and women to a similar degree | 0 | 24 (40.7%) | 8 (44.4%) |
| Other/neither | 0 | 24 (40.7%) | 6 (33.3%) |

**Table S1. Frequencies of Biological Sex, Gender Identity, and Gender of Preferred Sexual Partner Across Gender/Sexual Identity Groups**
